# QTc interval evaluation in patients with right bundle branch block or bifascicular blocks

**DOI:** 10.1002/clc.23389

**Published:** 2020-05-19

**Authors:** Damir Erkapic, Gerrit Frommeyer, Niklas Brettner, Korkut Sözener, Harry J. G. M. Crijns, Melchior Seyfarth, Christian W. Hamm, Harilaos Bogossian

**Affiliations:** ^1^ Diakonie Klinikum Siegen Department of Cardiology and Electrophysiology Siegen Germany; ^2^ Department of Cardiology and Angiology University Clinic of Gießen, Medical Clinic I Gießen Germany; ^3^ Clinic for Cardiology II – Electrophysiology University Clinic of Münster Münster Germany; ^4^ Department of Cardiology, Maastricht University Medical Center (MUMC+) and Cardiovascular Research, Institute Maastricht (CARIM) Maastricht The Netherlands; ^5^ Department of Cardiology Helios Klinikum Wuppertal Wuppertal Germany; ^6^ Department of Cardiology University Witten/Herdecke Witten Germany; ^7^ Department of Cardiology and Rhythmology Ev. Krankenhaus Hagen Hagen Germany

**Keywords:** bifascicular block, Bogossian formula, long QT, QT formula, QT prolongation, right bundle branch block

## Abstract

**Background:**

The right bundle branch block (RBBB) and the bifascicular blocks affect QRS duration in the right precordial leads, which are usually used for QT interval determination. Up to now, there is no clear recommendation how to determine QT interval in patients with RBBB or bifascicular block.

**Hypothesis:**

The hypothesis of the present study was to evaluate the feasibility of a simple formula for RBBB and bifascicular block correction, thereby making it easier to determine the QTc interval.

**Methods:**

In patients with intrinsic QRS duration <120 ms, artificial RBBB with either left posterior (LPFB) or left anterior fascicular block (LAFB), created by left ventricular pacing maneuvers, were corrected using the Bogossian formula (QTm) and afterward were heart rate corrected (QTmc). Heart rate correction was performed using different heart rate formulas in comparison to each other. The QTmc intervals were compared in each patient with the QTc interval during intrinsic rhythm.

**Results:**

A total of scheduled 71 patients were included in this prospective multicenter observational comparative study. Compared to intrinsic QTc interval, the mean ΔQTmc interval by combination of the Bogossian and the Hodge formulas was −3 ± 24 ms in RBBB + LPFB (*P* = .44) and −6 ± 25 ms in RBBB + LAFB (*P* = .15). The Bogossian formula showed a significant deviation from the actual QTc interval with both the Bazett and the Fridericia formulas.

**Conclusion:**

In combination with the Hodge formula, the Boggosian formula delivered the best results in comparing the true QTc interval in narrow QRS with the QTmc interval in the presence of a bifascicular block.

## 1 INTRODUCTION

**FIGURE 1:**
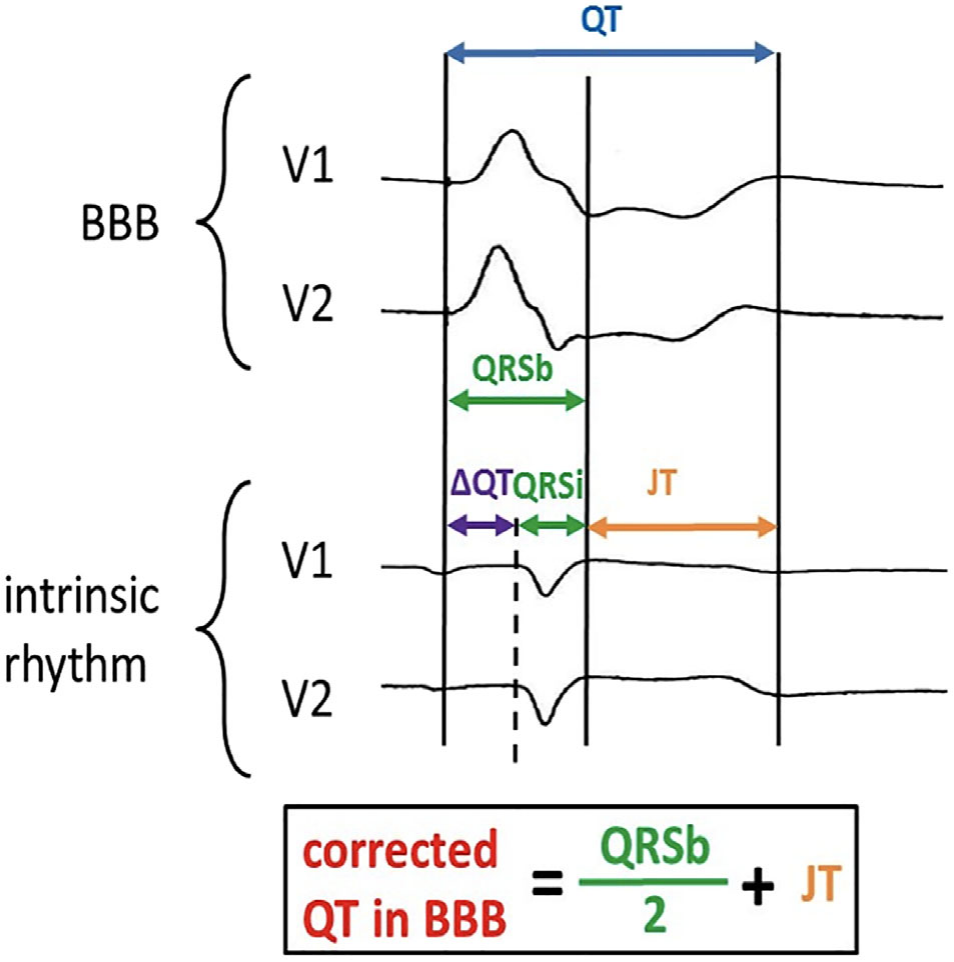
Utilization of the simplified Bogossian formula for bundle branch block correction and “true” QT interval evaluation. BBB, bundle branch block; QRSb: QRS in the presence of BBB; QRSi: intrinsic QRS in the absence of BBB; ΔQT: deviation of QT in the presence of BBB minus QT in the absence of BBB

Evaluation of the QTc interval is an important diagnostic tool in clinical practice to identify patients at high risk for ventricular tachycardia and sudden cardiac death.^1^ The presence of a bundle branch block (BBB) represents a particular challenge in properly measuring the QTc interval.^2^ Following international recommendations, QT interval should be measured in leads showing the longest QT interval, which is usually in right precordial leads.^3^ In presence of a right bundle branch block (RBBB) or a bifascicular block, these leads are strongest affected by conduction delay and therefore hamper adequate measurement. In 2014, a new formula for evaluation of the QT interval in patients with left bundle branch block (LBBB) was reported (Figure 1).^4^ Application of this so‐called “Bogossian formula,” in combination with the Bazett formula for heart rate correction showed to be a simple and reliable tool in clinical practice for QTc interval evaluation in patients with preserved or reduced left ventricular ejection fraction and LBBB.^4^, ^5^, ^6^, ^7^ However, the importance of the Bogossian formula has never been evaluated in patients with RBBB or bifascicular block. Moreover, for QTc interval evaluation, the Bogossian formula has never been combined with Fridericia's or Hodge's formulas.

## 2 METHODS

Patients who underwent left sided electrophysiologic study, respectively, ablation therapy, were eligible to participate in this prospective, multicenter observational study. Patients were included who had an intrinsic QRS interval of <120 ms, were between 18 and 80 years of age, had preserved left ventricular function, no cardiac device, and no history of myocardial infarction or ablation therapy ≤3 months before recruitment. Informed consent was obtained from each patient. All procedures performed in studies involving human participants were in accordance with the ethical standards of the institutional research committee (Giessen, AZ.: 127/16) and with the 1964 Helsinki declaration and its later amendments or comparable ethical standards.

**FIGURE 2:**
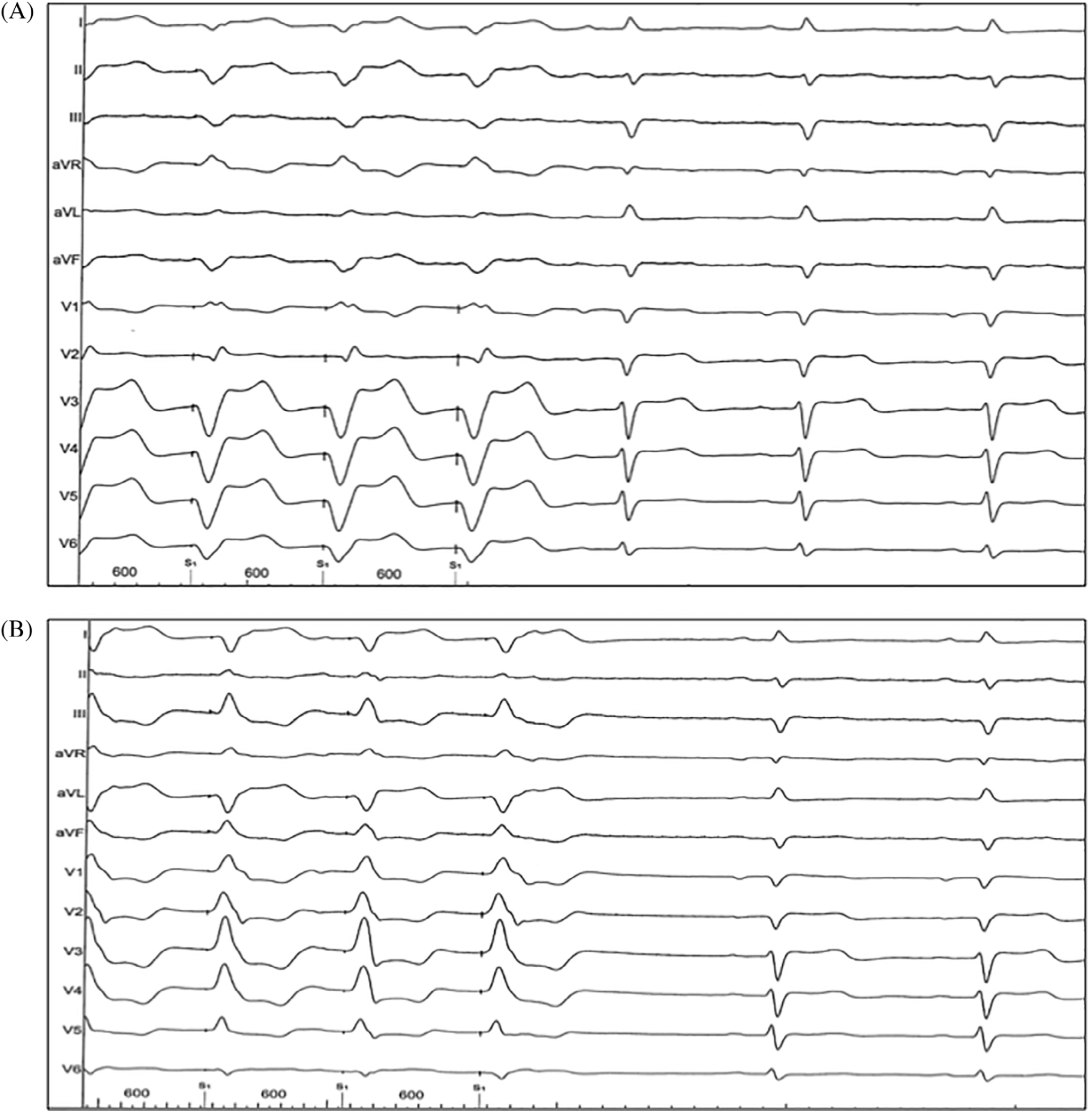
Bifascicular blocks created by left ventricular pacing. Twelve lead ECG examples with a paper speed of 50 mm/second. A, RBBB + LAFB ECG pattern during pacing (on the left side) and narrow QRS complexes during intrinsic rhythm (on the right side). B, RBBB + LPFB ECG pattern during pacing (on the left side) and narrow QRS complexes during intrinsic rhythm (on the right side). ECG, electrocardiogram; LAFB: left anterior fascicular block; LPFB, left posterior fascicular block; RBBB, right bundle branch block

All procedures were performed under conscious sedation with diazepam (Ratiopharm, Germany) and piritramide (Hameln, Germany). After a transseptal access with a SL‐1 sheath (Abbott, St. Paul, Minnesota) by modified Brockenbrough technique (BRK‐1, Abbott) followed by exchange of the SL‐1 sheath against a steerable Agilis sheath (Abbott) or a steerable FlexCath Advance sheath (Medtronic Inc., Mounds View, Minnesota), left ventricular pacing passed before scheduled ablation therapy. Pacing was performed with a steerable decapolar diagnostic catheter (Viacath, Biotronik, Germany) or a quadripolar TactiCath Contact Force catheter (Abbott), with a pacing rate above the intrinsic heart rate. To achieve a bifascicular block ECG pattern, pacing was performed in the region of the left anterior fascicle, resulting in a RBBB + left posterior fascicular block (LPFB) ECG pattern as well as in the region of the left posterior fascicle, resulting in a RBBB + left anterior fascicular block (LAFB) ECG pattern (Figure 2). All digital 12‐lead electrocardiograms were recorded using a Bard EP Mapping System (Boston Scientific, Marlborough, Massachusetts) at a speed of 50 mm/second. QT interval was measured on the lead presenting the longest interval. After RBBB, respectively, bifascicular block correction using the Bogossian formula, the QTc interval was evaluated as well with the Bazett formula as with the Fridericia and the Hodge formulas.^8^, ^9^, ^10^ The bundle branch block corrected QTc interval (QTmc) was compared in each patient with the QTc interval during intrinsic rhythm, measured with a tangent on the lead presenting the longest QT interval.^11^ In presence of atrial fibrillation, 10 consecutive RR intervals were averaged for the heart rate corrected QT interval. All RBBB corrections and QTc measurements were performed by two electrophysiologists (young electrophysiologist and experienced electrophysiologist) who worked independently from each other.

### 2.1 Statistical analysis

Continuous data are displayed as mean ± SD. Data were analyzed by employing a computerized database (Microsoft Excel 2010, Microsoft, Redmond, Washington) and were statistically evaluated using SPSS Software Release 23.0.0. The Shapiro‐Wilk test was employed to assess normal distribution. Differences between groups were determined by Student's unpaired *t*‐test. Differences were regarded significant when *P* < .05 (two‐sided).

## 3 RESULTS

A total of 71 patients who met inclusion criteria were enrolled in this prospective study. Patient characteristics were displayed in Table 1. The majority of patients (89%) were in sinus rhythm during electrophysiological examination. Pacing rates were 36 ± 18 bpm above intrinsic rhythm to achieve stable left ventricular capture. In intrinsic rhythm, mean heart rate was 69 ± 13 bpm with QRS duration of 97 ± 7 ms, JT interval of 328 ± 38 ms and QTc interval of 452 ± 28 ms using Bazett's formula, 442 ± 25 ms with Fridericia's formula, and 441 ± 24 ms with Hodge's formula, measured in leads V1 or V2, respectively. In both bifascicular blocks, longest QT interval was measured in lead V1 or V2. In RBBB with LAFB, QRS duration increased to 175 ± 21 ms, with QTmc interval of 466 ± 37 ms after QRS interval correction with the Bogossian formula and heart rate correction with the Bazett formula. Using Fridericia's formula QTmc interval was 425 ± 33 ms and with the Hodge formula 435 ± 26 ms. In RBBB with LPFB QRS duration increased to 179 ± 20 ms, with QTmc interval of 471 ± 31 ms, 431 ± 27 ms and 438 ± 23 ms after application of the Bazett, Fridericia, and Hodge formulas (Table 2). Compared to intrinsic rhythm, mean ΔQTmc using the combination of the Bogossian and Bazett formulas was 15 ± 33 ms in RBBB with LAFB (*P* = .01) and 20 ± 30 ms in RBBB with LPFB (*P* = .0002). When using the Bogossian and Fridericia formulas mean ΔQTmc was −17 ± 27 ms in RBBB with LAFB (*P* = .0006) and −11 ± 23 ms in RBBB with LPFB (*P* = .012). The combination of the Bogossian and the Hodge formulas showed a mean ΔQTmc of −6 ± 25 ms in RBBB with LAFB (*P* = .15) and −3 ± 24 ms in RBBB with LPFB (*P* = .44). The intrinsic JT interval was significantly changed by left ventricular stimulation from 328 ± 38 ms to 264 ms ± 35 ms in RBBB with LAFB and to 268 ± 31 ms in RBBB with LPFB (*P* = .0001, respectively). No significant difference was noted between the ECG measurements of both electrophysiologists (Tables 3 and 4).

**TABLE 1:** Baseline characteristics

	Patients (n = 71)
Age (years)	65 ± 11
Male gender	67%
BMI (kg/m^2^)	28 ± 4
Cardiac rhythm	
Sinus rhythm	89%
Atrial fibrillation	11%
LVEF (%)	59 ± 5
Coronary artery disease	32%
Hypertension	67%
Diabetes	13%
Renal dysfunction	7%
Previous TIA/Stroke	7%
Antiarrhythmic agents	
Class I	14%
Class II	75%
Class III	17%
Class IV	0%
ACE inhibitors/ARB	63%
Diuretics	26%
Electrophysiologic procedure	
PVC or idiopathic VT Ablation	19%
AP Ablation	1%
PVI	80%

*Note:* Data given as mean ± SD or in percentage.

Abbreviations: ACE, angiotensin converting enzyme; ARB, angiotensin receptor blockers; AP: accessory pathway; BMI, body mass index; LVEF, left ventricular ejection fraction; PVC, premature ventricular complex; PVI, pulmonary vein isolation; TIA, transient ischemic attack; VT, ventricular tachycardia.

**TABLE 2:** Comparison of mean values of ECG parameters after bifascicular block and heart rate correction using different formulas by experienced electrophysiologist

		RBBB + LAFB				RBBB + LPFB		
	Intrinsic	Paced	Corrected^a^	*P* value	Intrinsic	Paced	Corrected^a^	*P* value
HR (bpm)	69 ± 13	106 ± 18		.0001	69 ± 13	103 ± 20		.0001
QRS (ms)	97 ± 7	175 ± 21		.0001	97 ± 7	179 ± 20		.0001
JT (ms)	328 ± 38	264 ± 35		.0001	328 ± 38	268 ± 31		.0001
QT (ms)	425 ± 37	442 ± 42		.01	425 ± 37	445 ± 64		.022
QTc_B_ (ms)	452 ± 28		466 ± 37	.01	452 ± 28		471 ± 31	.0002
ΔQTmc_B_ (ms)			15 ± 33				20 ± 30	
QTc_F_ (ms)	442 ± 25		425 ± 33	.0006	442 ± 25		431 ± 27	.012
ΔQTmc_F_ (ms)			−17 ± 27				−11 ± 23	
QTc_H_ (ms)	441 ± 24		435 ± 26	**.15**	441 ± 24		438 ± 23	**.44**
ΔQTmc_H_ (ms)			−6 ± 25				−3 ± 24	

*Note:* Data given as mean ± SD. The values in bold are the only ones that show no statistically significant deviation from the actual QTc interval. This shows that the combination of the Hodge and Bogossian formulas is the preferred combination to determine the actual QTc interval.

Abbreviations: RBBB, right bundle branch block; LAFB, left anterior fascicular block; LPFB, left posterior fascicular block; HR, heart rate; QTc_B_, heart rate‐adjusted QT interval with Bazett's formula; QTc_F_, heart rate‐adjusted QT interval with Fridericia's formula; QTc_H_, heart rate‐adjusted QT interval with Hodge's formula; ΔQTmc_B_, deviation of QTc_B_ in presence of bifascicular block minus QTc_B_ in absence of bifascicular block (after modification using Bogossian's formula); ΔQTmc_F_, deviation of QTc_F_ in the presence of bifascicular block minus QTc_F_ in absence of bifascicular block (after modification using Bogossian's formula); ΔQTmc_H_, deviation of QTc_H_ in the presence of bifascicular block minus QTc_H_ in the absence of bifascicular block (after modification using Bogossian's formula).

a QRS interval during bifaszicular block, corrected to “narrow” QRS interval by Bogossian's formula and afterward heart rate corrected by Bazett's, Fridericia's, or Hodge's formula.

**TABLE 3:** Comparison of mean QTc intervals after bifascicular block correction by young and experienced electrophysiologist

	Intrinsic			RBBB + LAFB			RBBB + LPFB		
	QTmc_B_ (ms)	QTmc_F_ (ms)	QTmc_H_ (ms)	QTmc_B_ (ms)	QTmc_F_ (ms)	QTmc_H_ (ms)	QTmc_B_ (ms)	QTmc_F_ (ms)	QTmc_H_ (ms)
Young EP	452 ± 28	443 ± 21	442 ± 20	471 ± 35	429 ± 30	438 ± 25	471 ± 31	431 ± 27	438 ± 24
Experienced EP	452 ± 28	442 ± 25	441 ± 24	466 ± 37	425 ± 33	435 ± 26	471 ± 31	431 ± 27	438 ± 23
*P* value	1	.82	.78	.40	.44	.48	1	1	1

*Note:* Data given as mean ± SD.

Abbreviations: EP, electrophysiologist; LAFB, left anterior fascicular block; LPFB, left posterior fascicular block; QTmc_B_, bifascicular block affected QRS interval corrected with Bogossian's formula and afterward heart rate‐adjusted QT interval with Bazett's formula; QTmc_F_, bifascicular block affected QRS interval corrected with Bogossian's formula and afterward heart rate‐adjusted QT interval with Fridericia's formula; QTmc_H_, bifascicular block affected QRS interval corrected with Bogossian's formula and afterward heart rate‐adjusted QT interval with Hodge's formula; RBBB, right bundle branch block.

**TABLE 4:** Comparison of mean ΔQTmc intervals after bifascicular block and heart rate correction by young and experienced electrophysiologist

	RBBB + LAFB			RBBB + LPFB		
	ΔQTmc_B_ (ms)	ΔQTmc_F_ (ms)	ΔQTmc_H_ (ms)	ΔQTmc_B_ (ms)	ΔQTmc_F_ (ms)	ΔQTmc_H_ (ms)
Young EP	19 ± 35	−13 ± 28	−3 ± 25	19 ± 32	−12 ± 25	−4 ± 24
Experienced EP	15 ± 33	−17 ± 27	−6 ± 25	20 ± 30	−11 ± 23	−3 ± 24
*P* value	.48	.38	.47	.85	.80	.80

*Note:* Data given as mean ± SD.

Abbreviations: EP: electrophysiologist; LAFB: left anterior fascicular block; LPFB: left posterior fascicular block; ΔQTmcB: deviation of QTcBAZETT in presence of bifascicular block minus QTcBAZETT in absence of bifascicular block (after modification using Bogossian's formula); ΔQTmcF: deviation of QTcFRIDERICIA in presence of bifascicular block minus QTcFRIDERICIA in absence of bifascicular block (after modification using Bogossian's formula); ΔQTmcH: deviation of QTcHODGE in presence of bifascicular block minus QTcHODGE in absence of bifascicular block (after modification using Bogossian's formula); RBBB: right bundle branch block.

## 4 DISCUSSION

To best of our knowledge, this is the first prospective multicenter study to assess QTc interval evaluation in patients with bifascicular blocks. This trial shows that bifascicular blocks can be simple and reliable corrected by using the Bogossian Formula, followed by an acceptable heart rate‐adjusted QT interval determination using the Hodge formula.

Bundle branch blocks are frequent ECG findings, especially in the presence of a structural heart disease. Right bundle branch and/or bifascicular blocks increase with age and affect approximately 1% of the general population.^12^, ^13^ Furthermore, in patients hospitalized due to syncopes, bifascicular blocks can be detected with a frequency of up to 8%.^14^, ^15^, ^16^

In BBB, the cardiac conduction disorder is mainly affected by delay in depolarization, visible on ECG as extension of the QRS duration. Therefore, some authors recommend to use the JT interval for QT determination, due to its independence of QRS wideness.^17^, ^18^ However, the JT interval is rarely used in clinical practice, perhaps due to well‐established automated ECG measurements with rate correction of the QT interval. Nevertheless, these automated ECG measurements are not suitable in the presence of a BBB. In our study, the JT interval during left ventricular pacing was significantly shorter compared to the JT interval during intrinsic rhythm. This is explained by the required high left ventricular stimulation rate due to observed heightened mechano‐electrical feedback compared to right ventricular pacing in our initial trial.^4^ It is known that a higher heart rate results in an increasing difference between intrinsic and heart rate corrected QT interval.^6^, ^7^, ^19^ While the Bazett formula leaves a strong positive residual correlation with heart rate, the Fridericia formula leaves a negative correlation.^3^ For heart rate‐dependent QT correction using Bazett's or Fridericia's formulas deviations of more than 20 ms are considered to be possible.^3^, ^20^ In our study, the use of the Bazett and Fridericia formulas in combination with the Bogossian formula for correction of the QT interval in the presence of bifascicular blocks displayed the same mean deviations. The observed overestimation with the Bazett formula of up to 20 ms, in the current study, is in line with our previous results of LBBB correction using the combination of the Bogossian and the Bazett formulas.^6^, ^7^ To avoid a potential false positive QT prolongation using the Bazett formula or possible false negative QT prolongation using the Fridericia formula, the Hodge formula seems to be the best one for the combination with the Bogossian formula in assessing the “true” QTc interval in RBBB with additional block of one of the left ventricular fascicles.

Although this study involves artificially created bifascicular blocks, similar to those initially shown with the LBBB, the experimentally validated Bogossian formula has already shown that it holds true in clinical setting.^5^

The present study examined the feasibility of correcting RBBB and bifascicular blocks. Although we did not create an isolated RBBB ECG pattern with our stimulation maneuvers, it could be shown, that the longest QT interval could be measured in the leads V1 or V2. Since these leads are also most affected in isolated RBBB, it can be assumed that the Bogossian formula can also be applied to this form of bundle branch block.

An adequate evaluation of the QT interval is anything but trivial. Even in experienced colleagues, a correct hit rate of only 60% has been described in the past.^21^ In case of a BBB, the evaluation is made even more difficult. Therefore, standardized, simple and investigator‐friendly methods are needed to facilitate the evaluation. Since there was no significant difference in QTc interval measurements by the two independent operators in this trial, the Bogossian formula shows to have the capability to facilitate QT interval determination in patients with bifascicular block and probably in RBBB too.

## 5 CONCLUSION

In the present study, the Bogossian formula in combination with the Hodge formula delivered the best results in comparing the “true” QTc interval in narrow QRS with the corrected QT‐interval in the presence of bifascicular blocks, which were created by left ventricular pacing. The utilization of these formulas and the reproducibility of the results were independent of the operator. Therefore, the Bogossian formula seems to be a simple and investigator‐friendly method to correct BBB and to facilitate the determination of QTc interval, not only in patients with LBBB as previously reported, but also in RBBB with or without additional block of one of the left ventricular fascicles.

## CONFLICT OF INTEREST

The authors declare no potential conflict of interests

## AUTHOR CONTRIBUTIONS

All authors contributed to the study conception and design. Material preparation, data collection and analysis were performed by Damir Erkapic, Harilaos Bogossian, Niklas Brettner, and Geritt Frommeyer. The first draft of the manuscript was written by Damir Erkapic and all authors commented on previous versions of the manuscript. All authors read and approved the final manuscript.
